# Validity and reliability of a food frequency questionnaire for assessing dietary intake among Shanghai residents

**DOI:** 10.1186/s12937-019-0454-2

**Published:** 2019-05-23

**Authors:** Jiajie Zang, Baozhang Luo, Suying Chang, Shan Jin, Chengdi Shan, Lifang Ma, Zhenni Zhu, Changyi Guo, Shurong Zou, Xiaodong Jia, Fan Wu

**Affiliations:** 1 Shanghai Municipal Center for Disease Control and Prevention, Shanghai, 200336 China; 2 United Nations Children’s Fund Office for China, Beijing, China; 3 Huangpu District Center for Disease Control and Prevention, Shanghai, 200336 China; 4 Shanghai Institutes for Prevention Medicine, Shanghai, 200336 China

**Keywords:** Dietary records, Energy intake, Epidemiological studies, Food frequency questionnaire (FFQ), Reliability, Reproducibility, Validity

## Abstract

**Background:**

Few localized food frequency questionnaires (FFQ) have been developed and used in Chinese nutrition surveys despite China’s large population and diverse dietary habits.

**Method:**

We analyzed data collected in two waves (six months apart) of the Shanghai Diet and Health Study in 2012–2013, from 1623 Shanghai residents (798 men and 825 women) older than 18 years. The results of 3-day 24-h dietary recalls (HDR) plus condiment weighing were used to evaluate the validity and reliability of the SDHS FFQ.

**Results:**

The median and first and third quartiles for energy intake (in kcal) derived from the FFQ1 and FFQ2 were 1566.5 (1310.1–1869.6) and 1561.9 (1280.2–1838.4), respectively, of which protein (in g) was 54.3 (42.5–65.8) and 52.9 (42.4–64.5), fat (in g) was 49.8 (37.2–64.7) and 47.9 (34.9–61.9), and carbohydrates (in g) was 227.3 (180.8–277.9) and 228.1 (182.2–275.2) in the reliability analysis. The median and first and third quartiles for energy-intake differences between the FFQ1 and the 3-day 24-HDR with condiment weighing was 59.3 (− 255.5–341.6), of which protein was − 5.2 (− 18.7–7.8) and fat was − 11.2 (− 30.8–5.3). The adjusted Spearman’s correlations were 0.33–0.77 for validity and 0.46–0.79 for reliability. The intra-class correlation coefficients exceeded 0.46 (validity) and 0.47 (reliability) for macronutrient intake. The consistency between the same and adjacent quartiles was approximately 80% for various nutrients.

**Conclusion:**

The reliability and comparative validity of the SDHS FFQ is similar to FFQs that are used worldwide.

**Electronic supplementary material:**

The online version of this article (10.1186/s12937-019-0454-2) contains supplementary material, which is available to authorized users.

## Background

Although 24-h dietary recall (24-HDR) and dietary records with weighed foods have been used to measure usual dietary intake, the resource burden and economic constraints of these methods make them unsuitable for most large-scale studies [1, 2]. Short-term recalls and dietary records are expensive and unrepresentative of usual intake and therefore, inadequate for the assessment of past dietary intake. The food frequency questionnaire (FFQ) is the most commonly used instrument to assess past dietary intake in epidemiological studies on the relationship between dietary factors and diseases, primarily because of its low cost and ability to capture usual dietary patterns [3].

Frequency data can explain much of the variation in dietary intake, and FFQs can provide sufficient accuracy to rank individuals in terms of risks for subsequent health outcomes. FFQs have been used in many studies to predict associations between dietary intake and disease-specific mortality and morbidity [4]. However, the performance of an FFQ depends on its sensitivity to cultures, ethnic backgrounds, geographical areas and differences in study populations. Thus, the validity and reliability of FFQs should be evaluated for use with diverse samples.

In 2012, a population-based cohort study in Shanghai, China was initiated. The Shanghai Diet and Health Study (SDHS) was designed and implemented by the government to investigate the nutritional status of Shanghai’s residents and food contaminants in Shanghai. It also aimed to examine how these factors affect health outcomes, given Shanghai’s rapid economic development and the introduction of considerable variations in diet and eating habits that may influence food intake [5–8]. The SDHS proposal included assessments of dietary intake of the cohort members at the baseline survey (spring, summer, fall and winter, 2012–2013), and every 5–10 years afterwards. The purpose of the population-based cohort study was to collect data on the dietary intake and diet transitions of Shanghai residents.

The FFQ that was used previously in different geographical areas for the National Nutrition and Health Survey was also used in Shanghai [9]. However, substantial dietary discrepancies among different areas due to different dietary habits and traditions were found. Therefore, the SDHS developed a localized FFQ based on similar FFQs and local dietary habits. At baseline and follow-up, we collected dietary data using both the 24-HDR plus condiment weighing and FFQs in four waves (seasons). The aim of this research was to evaluate the validity and reliability of a 134-item quantitative FFQ. The instrument was designed to capture the usual dietary intake of Shanghai participants, and therefore, consisted of foods commonly consumed by Shanghai residents. The validated FFQ can provide an alternative way to capture dietary intake in future follow-ups and it can be used in related studies.

## Methods

The SDHS is an ongoing open-cohort study that was initiated in 2012. It was designed and implemented by the Shanghai government as a prospective examination of food consumption, energy and nutrient intake, and food contaminants in Shanghai, and their effects on the health of its residents. Given Shanghai’s rapid economic growth [5], the consumption patterns and eating habits of Shanghai residents have undergone great changes, which are likely to influence food intake and health outcomes. The study’s design was explained in the published literature [5–8]. Four waves of the SDHS were implemented during its first 2 years. Data were collected during May–June 2012 (spring), August–September 2013 (summer), November–December 2012 (fall) and January–February 2013 (winter). All the participants completed a 3-day 24-HDR and condiment weighing and an FFQ in each wave (season) [5–8].

### Development of the FFQ

The SDHS’s FFQ was developed using similar dietary questionnaires from several epidemiological studies conducted in Shanghai, the 2010–2013 nation-wide nutrition survey conducted in China [10–16] and updated information from Shanghai’s 2010–2011 dietary survey. The FFQ was designed for use by trained interviewers to collect information from Shanghai adults about their dietary intake during the previous 3 months. It is a 134-item quantitative FFQ with three parts consisting of supplementary questions regarding eating-out frequency, cooking oil and condiments, and eating habits. A total of 123 food items and food groups are included in the questionnaire, which represent approximately 95% of the most commonly consumed foods in Shanghai in 2011.

The FFQ food groups include staples, beans, vegetables, fungus, algae, fruit, dairy foods, meat, aquatic products, eggs, sweets and snacks, beverages and condiments. First, the participants were asked to report whether they consumed each food and food group. If they responded yes, they were asked to state how many times per day, week, month or year. The interviewer also asked participants about their average amount of intake for each item, each time it was consumed. The interviewers read aloud the standard portion size of each food item for every question. Visual aids relating to the standard portion sizes were shown to the participants.

Data pertaining to oils and condiments were obtained by inquiring about how many grams of the oils and other condiments were consumed by the entire family during the previous month, and how many family members consumed the condiments at home.

### Dietary validation analysis

Data from a sample of 1623 participants older than 18 years of age from the SDHS were included in the validity study. They completed the 134-item FFQ and a 3-day 24-HDR plus condiment weighing in all four waves (hereafter, “3-day 24-HDR plus condiment weighing” will be referred to as “24-HDR” for concision). Household condiment consumption (such as edible oils, salt, sauces, etc.) was determined by weighing all food consumed by the household over three consecutive days. Three-day 24 h recalls were done on three consecutive days to match with the weighing. It was determined by examining changes in inventory from the beginning to the end of each day, in combination with a weighing and measuring technique. All condiments remaining after the last meal before initiation of the survey were weighed and recorded. All purchases and wasted condiments were also recorded. At the end of the survey, all remaining condiments were again weighed and recorded. Data from two seasons (approximately 6 months apart) were used in this validity study. Wave 1 was conducted from January to February in 2013 and wave 2 from August to September 2013.

### Nutrient calculations

The nutrient database for the FFQ was developed in accordance with the constituent ratio of the amount of each food listed in the same item using representative 24-HDR data. For example, the citrus fruit group included oranges, tangerines, pomelos, and citrus gonggan. First, we calculated the amount of each food in the food group based on data obtained in the 24-HDR during each of the four waves of the study. Based on the constituent ratio of the amount of the top 10 foods weighted by their constituent ratios, the converted food composition database was recalculated for each food group.

The frequency of food intake was converted to the number of times consumed “per day” and multiplied by the amount of intake (g) to obtain the daily dietary intake of each food group. The amounts of oils and other condiments reported in the FFQ for the entire family was divided by the number of family members, and then, divided by the proportion of meals consumed at home, and converted to daily intake. The amount of the food intake per day was entered into the FFQ nutrient database. Daily totals for energy and nutrients were calculated, followed by macronutrient intake as a percentage of energy.

Data obtained from the 24-HDRs and the weighing of condiments were converted to the amount of each food item consumed per person per day. Then, the data on nutrients from the 24-HDR were analyzed using a food composition table developed by the Chinese Nutrition Society.

### Statistical analysis

Medians and interquartile ranges were calculated for all nutrients because the data for most of them were not distributed normally. The Wilcoxon signed-rank test was used to examine absolute differences between the FFQ and 24-HDR and the FFQ1 and FFQ2. Reproducibility was evaluated using all participants’ data from both of the FFQs by comparing the two rounds, using adjusted Spearman’s correlations. Comparative validity was assessed using adjusted Spearman’s correlations, and the Bland-Altman analysis [17] was used to examine differences between the FFQ and the 24-HDR. To examine the FFQ’s reliability and validity, participants were classified into quartiles based on the distributions of the data on their energy and macronutrient intake from the results of the FFQ and the reference method. Similar proportions of participants were classified into the same, adjacent or extreme quartiles. Correlations were used to detect linear relationships between the variables. Bland-Altman plots are used to evaluate agreement between two different measurements to determine the precision of one method compared with a reference method, in this study, to compare the FFQ with the 24-HDR. Quartile agreement was also used to assess quartile consistency. The results were considered statistically significant at a 0.05 level (two-tailed). Statistical analyses were performed with SAS software, version 9.4 (SAS Institute Inc., Cary, NC).

## Results

General characteristics of the participants (e.g., age, marital status, occupation, education, weight, family income, and region) are presented in Table 1. Data from 1623 participants, including 798 men and 825 women, were analyzed. More than half of the men and 0.6% of the women were current smokers. Alcohol consumption was reported by 33.7% of the men and 5.2% of the women.

**Table 1:** The characteristics of the included participants

Characteristics	Men		Women		All	
	n	% of Sub-group	n	% of Sub-group	n	% of Sub-group
Age group (years)						
15–44	230	28.8	233	28.2	463	28.5
45–59	279	35.0	298	36.1	576	35.5
≥ 60	289	36.2	294	35.7	583	35.9
Marital Status						
Married	653	81.8	639	77.5	1291	79.6
Other marital status	145	18.2	185	22.5	330	20.4
Occupation						
Professional job	198	24.8	126	15.3	324	20.0
Labor job	101	12.7	76	9.3	178	10.9
0Others	499	62.5	621	75.4	1120	69.1
Years of education						
≤ 6 years	165	20.7	219	26.6	384	23.7
7–9 years	248	31.0	261	31.7	509	31.4
10–12 years	205	25.7	189	22.9	394	24.3
> 12 years	180	22.6	155	18.8	335	20.7
Weight Status						
Underweight	18	2.2	30	3.6	48	2.9
Normal	304	38.1	368	44.6	671	41.4
Overweight	285	35.7	205	24.9	490	30.2
Obese	63	7.8	38	4.6	100	6.2
Non-reported	129	16.1	184	22.3	313	19.3
Smoker						
No	370	46.4	819	99.4	1189	73.3
Yes	428	53.6	5	0.6	433	26.7
Drinker						
No	474	59.4	756	91.8	1230	75.9
Yes	269	33.7	43	5.2	311	19.2
Non-reported	55	6.9	25	3.0	80	4.9
Family Income						
< 20,000 RMB/person	38	4.7	50	6.1	88	5.4
20,000–50,000 RMB/person	249	31.2	258	31.3	506	31.2
> 50,000 RMB/person	193	24.1	213	25.8	405	25.0
Non-reported	319	40.0	304	36.9	623	38.4
Region						
Urban	363	45.5	388	47.0	750	46.3
Suburban	200	25.1	209	25.3	409	25.2
Rural	235	29.5	228	27.6	463	28.5

The median, first, and third quartiles for the energy and macronutrient intake were estimated using two FFQs and 24-HDRs. Differences between the FFQ1 and 24-HDR are presented in Table 2. The median and first and third quartiles for energy intake (kcal) derived from the FFQ1 and FFQ2 were 1566.5 kcal (1310.1–1869.6 kcal), and 1561.9 kcal (1280.2–1838.4 kcal), respectively, of which protein was 54.3 g (42.5–65.8 g) and 52.9 g (42.4–64.5 g), fat was 49.8 g (37.2–64.7 g) and 47.9 g (34.9–61.9 g), and carbohydrates was 227.3 g (180.8–277.9 g) and 228.1 g (182.2–275.2 g) in the reproducibility analysis. In general, there was no significant difference between waves 1 and 2 of the FFQs. The median and first and third quartiles for energy-intake differences between the FFQ1 and the 24-HDR was 59.3 kcal (− 255.5–341.6 kcal), of which protein was − 5.2 g (− 18.7–7.8 g) and fat was − 11.2 g (− 30.8–5.3 g).

**Table 2:** Median nutrient intake between two FFQ and average of 24-HDR among participants in Shanghai Diet and Health Study

Energy and nutrients	FFQ1		FFQ2		24-HDR		Wilcoxon ranked test *P* values	
	Median	25–75th percentile	Median	25–75th percentile	Median	25–75th percentile	FFQ1 VS FFQ2	24-HDR FFQ2
Energy(kcal)	1566.5	1310.1–1869.6	1561.9	1280.2–1838.4	1515.2	1222.2–1819.7	0.16	0.06
Protein(g)	54.3	42.5–65.8	52.9	42.4–64.5	58.7	45.9–74.2	0.07	0.08
Fat(g)	49.8	37.2–64.7	47.9	34.9–61.9	60.9	46.6–78.8	0.13	< 0.01
Carbohydrate (g)	227.3	180.8–277.9	228.1	182.2–275.2	173.3	134.5–220.3	0.12	< 0.01
Protein(% energy)	13.7	12.0–15.4	13.6	11.9–15.4	15.6	13.3–18.1	0.21	0.05
Fat(% energy)	28.6	22.7–34.6	27.6	21.3–33.6	37.3	30.2–45.4	0.17	< 0.01
Carbohydrate (% energy)	58.5	52.6–64.4	59.5	53.2–65.9	46.8	40.1–53.9	0.10	< 0.01
Vitamin A(μg)	300.3	214.9–407.8	301.3	213.8–400.2	361.6	242.1–508	0.12	< 0.01
Carotene(μg)	936.0	640.6–1359.2	951.0	634.2–1285	1244.0	704.3–2016.8	< 0.01	< 0.01
Retinol(μg)	133.9	83.4–191.2	129.1	78.3–189.6	131.1	84.3–189	0.05	0.13
Vitamin D(IU)	12.0	4.2–23.8	6.0	2.2–13.6	0.0	0.00	< 0.01	< 0.01
Vitamin E(mg)	19.7	13.1–26.4	17.8	10.2–24.9	22.2	15.6–31.6	0.03	0.58
Vitamin K(μg)	8.1	4.3–13.7	12.5	6.4–21.5	0.0	0.00	0.01	< 0.01
Thiamine(mg)	0.6	0.5–0.8	0.6	0.5–0.8	0.7	0.5–0.9	0.12	0.06
Riboflavin(mg)	0.7	0.5–1	0.7	0.5–0.9	0.8	0.6–1	0.08	0.07
Niacin(mg)	10.5	8.3–13.1	10.8	8.7–13.3	13.1	10.1–16.8	0.24	0.21
Folate (μg)	9.9	6.1–14.9	10.7	7.2–16.8	2.8	0.1–16.2	0.14	< 0.01
Biotin(μg)	1.1	0.5–1.8	0.8	0.4–1.4	0.3	0.0–0.9	0.06	0.01
Choline(mg)	6.6	2.5–14.6	3.2	0.5–10	0.0	0.0–1.9	< 0.01	< 0.01
Vitamin C (mg)	46.9	32.1–63.7	47.4	32.2–63.8	58.0	37.8–83.2	0.19	0.06
Calcium (mg)	352.6	230.6–530	341.3	232.9–506.8	376.3	259.9–548.2	0.01	< 0.01
Phosphorus (mg)	786.8	602.5–966.4	761.4	602.6–949.7	818.8	650.7–994.3	< 0.01	< 0.01
Potassium (mg)	1386.3	1029.9–1781.1	1313.3	1001.3–1660.3	1495.2	1129.0–1924.2	< 0.01	< 0.01
Sodium (mg)	3622.8	1001.9–5115.5	2950.9	638.0–4721.8	3914.8	2809.7–5504.2	< 0.01	< 0.01
Magnesium (mg)	212.4	166.7–262.7	208.4	169.2–255.9	221.6	173.3–280.1	0.21	0.09
Iron (mg)	14.5	11.7–18.1	14.1	11.6–17.5	16.2	13.1–20.9	0.58	0.01
Zinc (mg)	8.5	6.9–10.2	8.5	7.0–10.3	8.5	6.7–10.4	0.20	0.61
Selenium(μg)	39.3	28.8–49.9	37.5	28.8–49.8	43.3	30.1–56.8	0.12	< 0.01

The adjusted Spearman’s correlations for reproducibility ranged from 0.46 to 0.79. The consistency between the same and adjacent quartiles was 80%. The intraclass correlation coefficient (ICC) between the FFQ1 and FFQ2 were 0.59, 0.71, 0.48, 0.47 for energy and macronutrients intake, and ranged from 0.34 to 0.72for micronutrient intake (Table 3).

**Table 3:** Spearman correlation, ICC and percentage agreement in quartile distribution of nutrient intake between the two FFQs, among participants in Shanghai Diet and Health Study

Energy and nutrients	Correlation	ICC	Percentage agreement			
			Same quartile	Adjacent quartile	One quartile apart	Opposite quartile
Energy(kcal)	0.71	0.59	36.8	41.5	19.1	2.6
Protein(g)	0.56	0.71	37.5	42.3	16.2	4
Fat(g)	0.69	0.48	30.6	40.8	21	7.6
Carbohydrate (g)	0.62	0.47	35.3	41.1	18.6	5
Protein(% energy)	0.72	0.55	31.9	38.3	22.8	7
Fat(% energy)	0.71	0.58	28.7	39.7	21.3	10.4
Carbohydrate (% energy)	0.71	0.55	28.2	39.2	22.8	9.8
Vitamin A(μg)	0.56	0.38	34	39.8	20	6.2
Carotene(μg)	0.58	0.51	34.1	39.5	20.1	6.3
Retinol(μg)	0.64	0.40	32.7	41.2	19.3	6.8
Vitamin D(IU)	0.69	0.56	13.3	53.3	26.7	6.7
Vitamin E(mg)	0.79	0.37	35.3	37.8	19.8	7.1
Vitamin K(μg)	0.66	0.40	27.3	33.8	27.7	11.3
Thiamine(mg)	0.57	0.54	33	42	19.5	5.5
Riboflavin(mg)	0.59	0.59	40	42.1	13.4	4.6
Niacin(mg)	0.46	0.45	34.6	40.1	19.6	5.7
Folate (μg)	0.63	0.68	25.3	38	26.7	10
Biotin(μg)	0.68	0.69	25.2	35.4	27.2	12.3
Choline(mg)	0.69	0.37	23.2	37.6	26	13.3
Vitamin C (mg)	0.56	0.45	34	42.4	18.5	5.1
Calcium (mg)	0.62	0.72	39.9	41.5	14.7	4
Phosphorus (mg)	0.61	0.34	39.4	41.7	14.6	4.3
Potassium (mg)	0.55	0.51	36.9	42.1	17	4.1
Sodium (mg)	0.74	0.58	29.4	42.8	20.1	7.7
Magnesium (mg)	0.53	0.53	36.8	40.8	17.2	5.2
Iron (mg)	0.49	0.45	35.4	44.3	15.8	4.6
Zinc (mg)	0.49	0.61	33.9	40.2	21.1	4.8
Selenium(μg)	0.6	0.70	39.1	39.7	17	4.2

Comparative validity was evaluated by calculating the correlation coefficients for the FFQ and 24-HDR, as shown in Table 4. The adjusted Spearman’s correlations for validity ranged from 0.33 to 0.77. The consistency between the same and adjacent quartiles was 80%.

**Table 4:** Spearman correlation and percentage agreement in quartile distribution of nutrient intake between second FFQ with average 24-HDR, among participants in Shanghai Diet and Health Study

Energy and nutrients	Correlation	ICC	Percentage agreement			
			Same quartile	Adjacent quartile	One quartile apart	Opposite quartile
Energy(kcal)	0.77	0.51	35.4	43.7	17.5	3.5
Protein(g)	0.45	0.64	38.7	41.6	17.7	2.1
Fat(g)	0.61	0.50	27.4	41.4	22.9	8.3
Carbohydrate (g)	0.39	0.46	35.1	41.7	19	4.2
Protein(% energy)	0.76	0.50	30.2	41.5	20.3	8
Fat(% energy)	0.76	0.54	25.3	39.5	24.3	10.9
Carbohydrate (% energy)	0.77	0.66	26.3	40.1	22.8	10.8
Vitamin A(μg)	0.66	0.40	33	39.2	20.8	7.1
Carotene(μg)	0.67	0.46	30.2	39.2	23.6	7.1
Retinol(μg)	0.7	0.53	33.6	40.6	19.7	6.1
Vitamin D(IU)	0.76	0.47	18.2	72.7		9.1
Vitamin E(mg)	0.74	0.53	33.1	38.3	20.1	8.5
Vitamin K(μg)	0.73	0.36	33.6	31.1	27.7	7.6
Thiamine(mg)	0.35	0.47	35.7	42.8	16.1	5.4
Riboflavin(mg)	0.63	0.50	41.3	41	15.4	2.3
Niacin(mg)	0.4	0.46	34.9	42.7	17.9	4.6
Folate (μg)	0.7	0.60	26.5	38	24.7	10.9
Biotin(μg)	0.75	0.63	24.8	37.9	24.3	13
Choline(mg)	0.77	0.41	24.9	40.3	21.6	13.3
Vitamin C (mg)	0.67	0.61	33.7	38.7	21.1	6.5
Calcium (mg)	0.68	0.62	40.6	40.7	15.9	2.8
Phosphorus (mg)	0.51	0.58	41.6	40.9	14.6	2.9
Potassium (mg)	0.62	0.51	39.4	41.7	15.7	3.2
Sodium (mg)	0.76	0.61	27.4	39.3	23.8	9.6
Magnesium (mg)	0.48	0.53	34	42	19.1	4.9
Iron (mg)	0.42	0.41	34.9	41.3	18.5	5.3
Zinc (mg)	0.33	0.61	36.8	39.1	19.8	4.2
Selenium(μg)	0.57	0.65	40.8	40	15.9	3.3

The median, and first and third quartiles for energy and macronutrient intake were estimated using the two FFQs and 24-HDRs. The validity and reproducibility, as measured by correlations are summarized in Tables 5 and 6. The validity and the reproducibility of most of the energy and nutrient intake among the men were higher than that of the women. When the data were analyzed by age group, we found the highest reproducibility in the group that was less than 45 years old; however, the highest validity was found in the group that was older than 60 years of age (Additional file 1).

**Table 5:** Median comparison of nutrient intake between two FFQ and average of 24-HDR among participants in Shanghai Diet and Health Study for men

Energy and nutrients	FFQ1		FFQ2		24-HDR		Correlation	
	Median	25–75th percentile	Median	25–75th percentile	Median	25–75th percentile	FFQ1 versus FFQ2	24-HDR FFQ2
Energy(kcal)	1627.9	1370.4–1947.5	1614.5	1292.2–1911.7	1625.4	1318.9–1945.8	0.75	0.80
Protein(g)	56.1	43.3–68.4	54.1	43.0–65.8	63.1	49.8–79.7	0.55	0.58
Fat(g)	51.6	38.5–65.1	47.5	33.7–62	64.4	48.8–83.8	0.62	0.55
Carbohydrate (g)	238.5	191.3–288.9	237.1	190.7–287	183.7	145.9–233.6	0.63	0.46
Protein(% energy)	13.9	12.1–15.7	13.7	11.9–15.5	15.8	13.3–18.4	0.76	0.80
Fat(% energy)	26.8	21.3–32.3	25.8	19.6–31.3	37.2	29.6–44.5	0.74	0.80
Carbohydrate (% energy)	60.1	54.6–66	61.4	55.5–67.6	46.3	39.8–53.8	0.74	0.80
Vitamin A(μg)	295.8	209.1–401.2	287.5	211.2–386.1	371.1	247.7–519.9	0.53	0.68
Carotene(μg)	933.1	593.5–1390.9	957.6	641.7–1269	1258.0	704.3–2047.1	0.54	0.69
Retinol(μg)	130.1	81.6–185.8	123.2	75.0–183.5	131.9	86.6–189.4	0.66	0.73
Vitamin D(IU)	11.8	4.2–22.4	5.6	1.7–12.7	0.0	0.00	0.73	0.80
Vitamin E(mg)	20.1	13.5–26.2	17.2	9.5–24.4	22.9	15.8–32.6	0.78	0.75
Vitamin K(μg)	8.2	4.3–16.1	12.5	8.0–21.5	0.0	0.00	0.70	0.74
Thiamine(mg)	0.7	0.5–0.8	0.7	0.5–0.8	0.8	0.5–1	0.55	0.39
Riboflavin(mg)	0.7	0.5–0.9	0.7	0.5–0.9	0.8	0.6–1	0.59	0.70
Niacin(mg)	10.9	8.9–13.8	11.4	9.0–13.8	14.6	11.2–18.6	0.42	0.43
Folate (μg)	9.5	5.4–14.4	10.3	7.1–15.8	2.9	0.1–14.6	0.62	0.73
Biotin(μg)	0.9	0.5–1.6	0.7	0.3–1.3	0.3	0.0–0.9	0.69	0.79
Choline(mg)	6.1	1.9–13.3	2.2	0.4–8.2	0.0	0.0–2	0.71	0.81
Vitamin C (mg)	45.4	30.6–62.6	47.0	32.2–62.6	58.5	37.6–86.1	0.52	0.71
Calcium (mg)	327.6	222.0–501.6	312.4	221.6–461	376.7	264.0–555.6	0.64	0.74
Phosphorus (mg)	807.4	608.2–999.4	764.7	609.4–963.8	860.5	694.9–1059.1	0.62	0.62
Potassium (mg)	1370.6	1029.9–1778.1	1294.7	983.5–1639.1	1551.6	1192.7–2006.4	0.52	0.64
Sodium (mg)	3860.5	1090.3–5134.4	2562.0	572.2–4499.2	4184.7	2918.8–5938.2	0.73	0.79
Magnesium (mg)	215.3	171.3–268.3	213.5	169.5–259.7	230.1	183.6–290.8	0.50	0.54
Iron (mg)	15.1	12.1–18.6	14.4	11.8–18	17.4	13.9–22.6	0.45	0.46
Zinc (mg)	8.9	7.2–10.6	8.7	7.1–10.5	9.3	7.4–11.3	0.49	0.44
Selenium(μg)	39.8	29.3–50.7	38.6	28.5–49.1	46.0	32.8–61.5	0.59	0.69

**Table 6:** Median comparison of nutrient intake between two FFQ and average of 24-HDR among participants in Shanghai Diet and Health Study for women

Energy and nutrients	FFQ1		FFQ2		24-HDR		Correlation	
	Median	25–75th percentile	Median	25–75th percentile	Median	25–75th percentile	FFQ1 versus FFQ2	24-HDR FFQ2
Energy(kcal)	1509.2	1246.5–1781.6	1523.0	1271.4–1749.7	1419.6	1151.6–1701.7	0.66	0.66
Protein(g)	52.4	41.0–64.1	51.7	42.1–63.6	54.5	42.6–67.7	0.56	0.28
Fat(g)	48.6	36.6–63.8	48.1	35.4–61.9	57.5	45.1–74.6	0.72	0.63
Carbohydrate (g)	215.7	171.4–269.3	218.3	176.9–264.5	162.3	126.6–204.6	0.60	0.32
Protein(% energy)	14.1	12.4–15.9	13.8	12.3–15.8	15.1	13.1–17.3	0.66	0.65
Fat(% energy)	27.6	21.4–34.1	27.4	21.3–34.1	40.2	33.3–47.2	0.67	0.66
Carbohydrate (% energy)	59.5	52.8–65.5	59.1	53.1–66	45.2	38.8–51.3	0.66	0.66
Vitamin A(μg)	307.2	220.0–411.2	308.6	216.8–412.9	352.5	236.5–496.6	0.55	0.51
Carotene(μg)	936.9	678.2–1336.5	947.8	628.6–1288.7	1199.6	704.8–1967.2	0.60	0.55
Retinol(μg)	137.6	86.5–196.6	134.0	82.2–196.2	131.1	81.1–187.9	0.58	0.56
Vitamin D(IU)	12.4	4.1–26.6	6.3	2.5–14.4	0.0	0.00	0.63	0.65
Vitamin E(mg)	19.5	12.8–26.5	18.3	10.8–25.5	21.2	15.2–31.1	0.77	0.68
Vitamin K(μg)	8.1	4.0–12.9	12.5	5.4–21.5	0.0	0.00	0.62	0.63
Thiamine(mg)	0.6	0.5–0.7	0.6	0.5–0.7	0.6	0.4–0.8	0.59	0.28
Riboflavin(mg)	0.7	0.5–1	0.7	0.5–0.9	0.7	0.5–0.9	0.51	0.47
Vitamin B6(mg)	0.0	0.00	0.0	0.00	0.0	0.00	0.59	0.59
Vitamin B12(μg)	0.0	0.00	0.0	0.00	0.0	0.00	0.66	0.65
Niacin(mg)	10.0	8.0–12.3	10.3	8.5–12.8	11.7	9.2–15.1	0.53	0.37
Folate (μg)	10.3	6.6–15.5	11.3	7.2–17.1	2.4	0.1–17.3	0.61	0.57
Biotin(μg)	1.2	0.6–1.8	0.9	0.4–1.5	0.3	0.0–0.8	0.64	0.62
Choline(mg)	8.0	3.0–16.4	4.2	0.7–11.3	0.0	0.0–1.7	0.64	0.63
Vitamin C (mg)	48.3	33.4–64.2	47.9	32.0–64.4	56.8	37.9–82.1	0.58	0.53
Calcium (mg)	376.6	245.3–561.8	366.8	250.2–538.1	374.5	255.7–545.8	0.54	0.49
Phosphorus (mg)	775.1	592.3–958.5	756.2	592.5–944.1	782.4	615.4–952.8	0.52	0.34
Potassium (mg)	1395.7	1028.9–1781.5	1332.5	1017.4–1669.9	1453.4	1075.5–1823.4	0.50	0.47
Sodium (mg)	3299.1	901.5–5089	3180.2	704.5–4843.4	3787.2	2716.7–5064.7	0.71	0.67
Magnesium (mg)	207.5	163.6–257.2	205.5	167.5–252.2	208.6	164.6–265.8	0.51	0.32
Iron (mg)	14.1	11.4–17.7	13.9	11.5–17.1	15.3	12.2–19.5	0.52	0.31
Zinc (mg)	8.2	6.6–9.9	8.2	6.9–10	7.8	6.2–9.5	0.49	0.20
Selenium(μg)	39.1	28.4–49.3	37.3	29.0–50.1	39.7	28.8–52.7	0.60	0.37

**Fig. 1:**
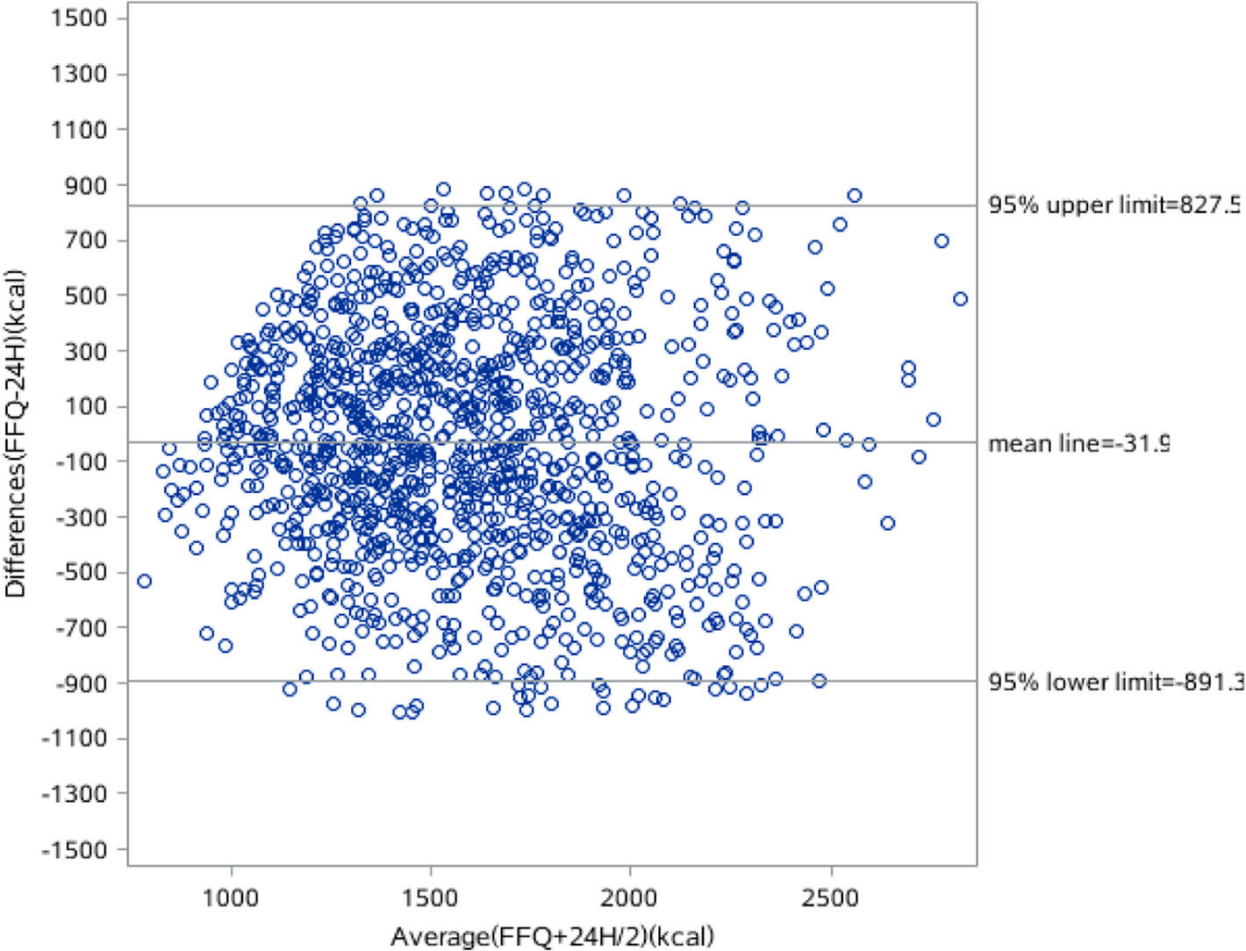
Bland-Alitman plot for total energy intake of Shanghai residents

**Fig. 2:**
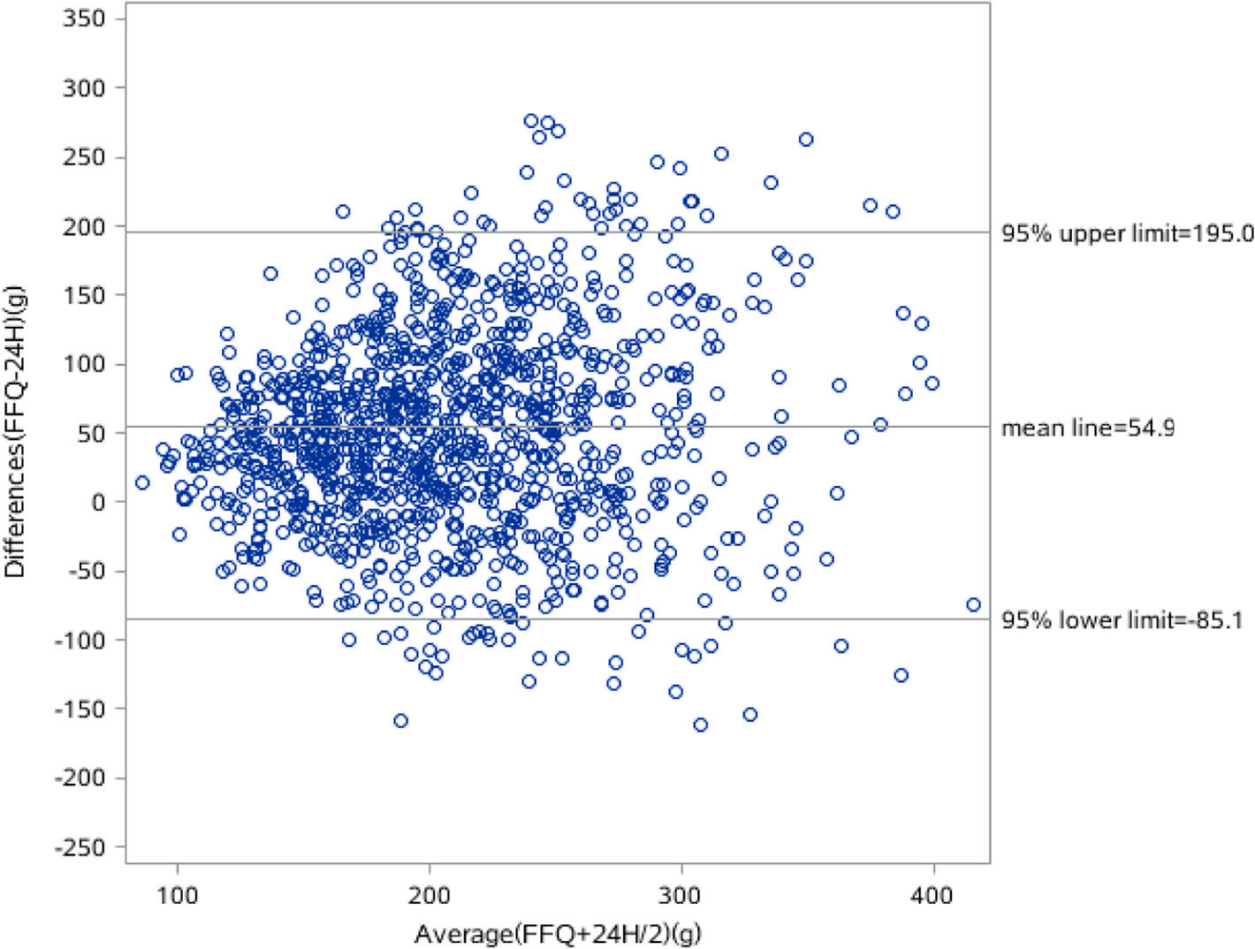
Bland-Alitman plot for carbohydrate intake of Shanghai residents

**Fig. 3:**
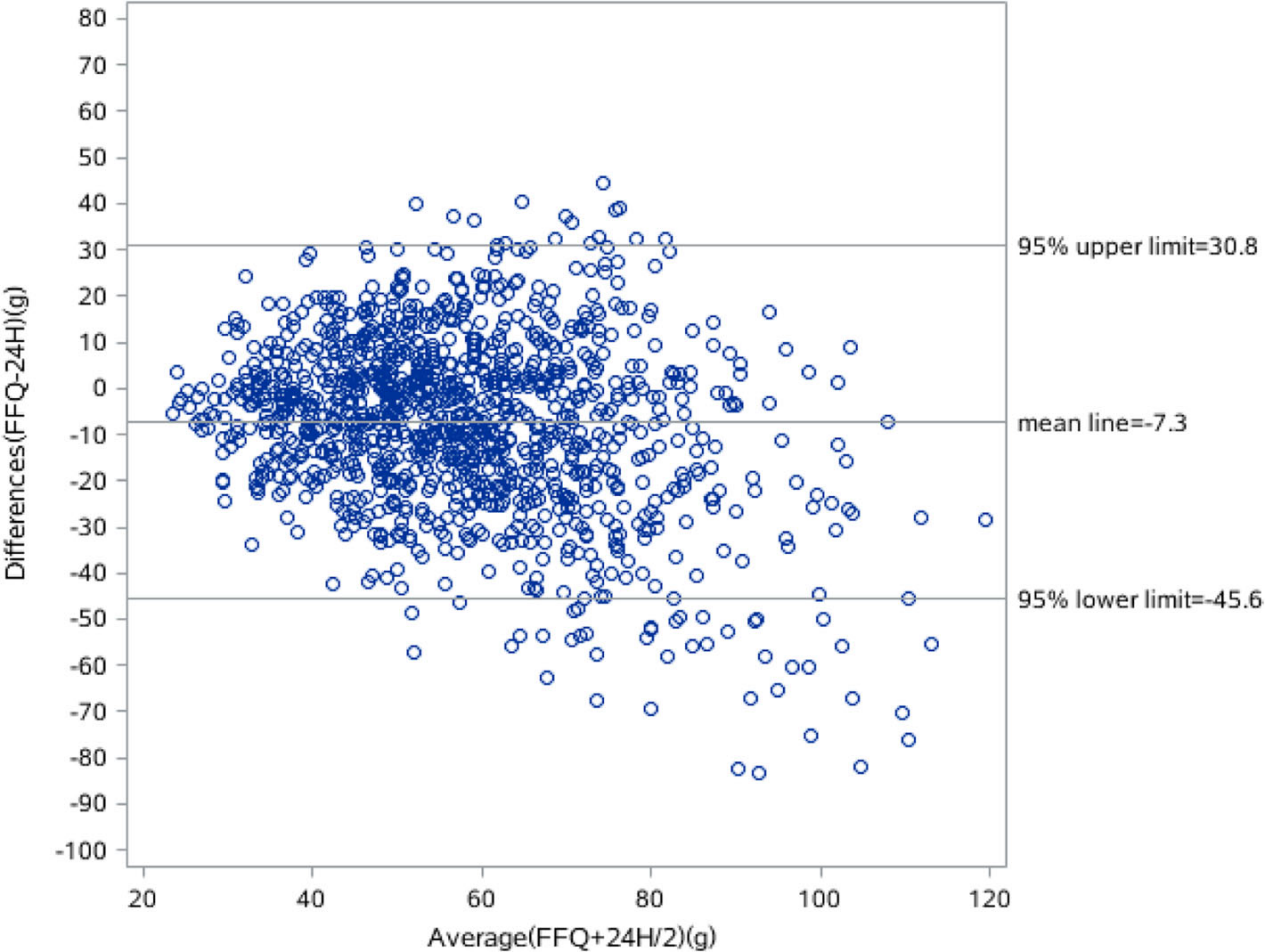
Bland-Alitman plot for protein intake of Shanghai residents

**Fig. 4:**
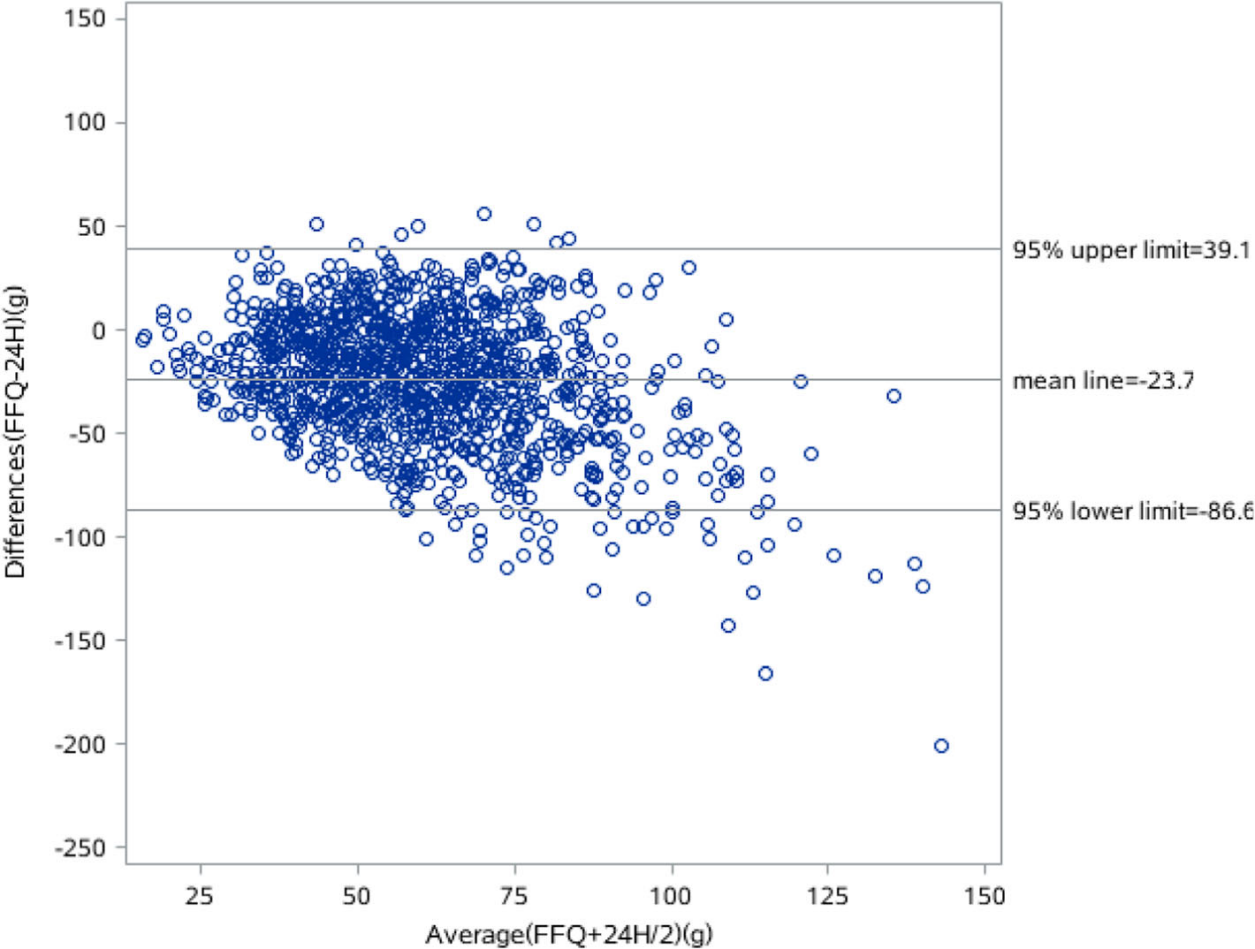
Bland-Alitman plot for fat intake of Shanghai residents

The results of the Bland-Altman analyses of the energy, protein, fat and carbohydrate intake are shown in Figs. 1, 2, 3 and 4. Differences in intake between the FFQ and the 24-HDR are plotted on the Y-axis and the mean intake derived from the two tools is presented on the X-axis. The mean difference and 95% lower and upper limits for energy intake was 31.9 kcal (− 891.3–827.5 kcal), of which protein intake was − 7.3 g (− 45.6–30.8 g), fat was − 23.7 g (− 86.6–39.1 g) and carbohydrate was 54.9 g (− 85.1.3–195 g).

## Discussion

The results indicate that the SDHS FFQ has comparative validity and reliability to the 24-HDR, and can be used to categorize major nutrients to determine their intake with relative accuracy among residents of Shanghai. This report describes the validity and reproducibility of a FFQ designed to capture Shanghai residents’ usual intake of nutrients. The reference method was the 24-HDR (i.e., 3-day 24-HDR and household condiment weighing), which was conducted at the beginning and the end of a 6-month period. We evaluated the performance of the FFQ by comparing the intake of nutrients reported using this instrument with the intake obtained using the 24-HDR.

The 24-HDR has been used in most nutrition studies in China [18]. The method provides accurate estimates of study participants’ usual dietary intake. However, this method is usually expensive, resource-intensive, and it yields only information collected over a short period [19]. FFQs have a lower respondent burden, are relatively inexpensive, do not require trained interviewers, and can be semi-automated using technology, making them practical for large epidemiological studies [20, 21]. Our study used a large representative population to test the validity and reliability of the FFQ by comparing it with the 24-HDR method. The FFQ was found to be useful in the nutrition survey.

In comparison with reproducibility and validity studies on other FFQs in the same population, we observed relatively higher correlation coefficients, indicating good reproducibility of our FFQ. Villegas et al. and Shu et al. have reported acceptable validity and reproducibility of an FFQ for the assessment of energy and nutrient intake in the Shanghai Men’s Health Study (SMHS) and Shanghai Women’s Health Study (SWHS). The ranges for Pearson’s correlations for validity and reproducibility were 0.33–0.58 and 0.38–0.53, respectively, for the SMHS, and 0.41–0.66 and 0.30–0.59, respectively, for the SWHS. The SMHS and SWHS reported differences between the FFQ and 24-HDR that ranged from − 21.3 to 31.8% and − 8.8 to 12.1, respectively [13, 15]. Our FFQ was developed based on the latest dietary data of Shanghai residents and the most frequently used FFQs in nation-wide surveys [18], whereas the FFQs used in the SMHS and SWHS were developed based on the most commonly consumed foods in urban Shanghai in 1996. Due to significant changes in dietary intake, China’s food supply has become much more diverse and “off-season” foods can be found in homes during most times of the year in developed cities, such as Shanghai. Our FFQ provides a better representation of the food items and food groups recently found in Shanghai. Moreover, our study collected data on oils, salt and other condiments in our FFQ, which provided dietary data that are more comprehensive than those reported in other studies. Subar et al. [22] reported scores ranging from 0.41 to 0.83 on the Diet History Questionnaire, 0.19 to 0.80 on the Block FFQ and 0.28 to 0.83 on the Willett FFQ using measurement error models adjusted for energy with the 24-HDR as the reference method. The findings from our study are consistent with their results.

Assuming the data obtained from the 24-HDR is close to participants’ “true” intake, we found that the SDHS FFQ can provide a better estimate of macronutrients, including energy and the contributions of protein, fat and carbohydrates, and other nutrients, such as cholesterol, pantothenate, choline, folate, Vitamin E, Biotin and Vitamin K. However, our FFQ did not perform as well on estimates of thiamine, zinc and niacin, which may be due to seasonal food differences between the FFQ and the 24-HDR.

The strength of our study is that we included more than 1.6 thousand randomly selected residents from all districts of Shanghai to provide the best representation of food intake of the Shanghai population compared to any other study conducted in the same area. Second, we developed our FFQ based on the latest local dietary survey and other FFQs in China with similar objectives to reveal the actual intake of this population. Third, we added condiment-related questions to our FFQ, which have not been analyzed with other samples. This change should provide dietary data that are more comprehensive than other FFQs. Fourth, we originally collected data four times during each of the four seasons and they were found to have validity and reliability for all four seasons. We found comparative validity and reliability among the seasons (Additional file 1: Tables S1-S4). This is the first study to examine the validity and reliability of a FFQ over four seasons. We assessed the FFQ’s validity and reliability by gender and age group and found differences among these groups.

This study has some limitations. First, the FFQ has been validated only as an interviewer-administered FFQ but not as a self-report questionnaire, Second, the FFQ evaluated in our study is a localized data-collection instrument that may be used to assess the local diets of populations from Shanghai or southeast China. Moreover, the results are not necessarily transferable to other populations because of regional variations in local foods [23, 24]. Each region of China should develop a localized FFQ in accordance with their specific dietary habits and traditions instead using a uniform FFQ from nationwide surveys. Validity and reproducibility may vary by gender and age; therefore, researchers should carefully recruit diverse groups. In conclusion, this study indicates that the SDHS FFQ can reliably and accurately measure the usual intake of major nutrients among residents of Shanghai.

## Conclusion

The reliability and comparative validity of the SDHS FFQ is similar to FFQs that are used worldwide.

## Additional file

Additional file 1: **Table S1.** Median and 25–75th percentile(Q1-Q3) of energy and nutrient intake of FFQs among seasons in Shanghai Diet and Health Study. **Table S2.** Median and 25–75th percentile(Q1-Q3) of energy and nutrient intake of 24-HDR among seasons in Shanghai Diet and Health Study. **Table S3.** Validity and reliability of the FFQs in different seasons. **Table S4.** Validity and reliability of the FFQs in different subgroups. (DOCX 39 kb)

## Abbreviations

24-HDR: 24-h dietary recalls
FFQ: Food frequency questionnaires
SDHS: Shanghai Diet and Health Study
SMHS: The Shanghai Men’s Health Study
SWHS: Shanghai Women’s Health Study
