# Supplementary material for: Validity and reliability of a food frequency questionnaire for assessing dietary intake among Shanghai residents
Source: Nutr J. 2019 May 23;18:30. doi: 10.1186/s12937-019-0454-2 (PMC6533674; doi:10.1186/s12937-019-0454-2)
Supplement: Supplementary file 1 — Table S1. Median and 25–75th percentile(Q1-Q3) of energy and nutrient intake of FFQs among seasons in Shanghai Diet and Health Study. Table S2. Median and 25–75th percentile(Q1-Q3) of energy and nutrient intake of 24-HDR among seasons in Shanghai Diet and Health Study. Table S3. Validity and reliability of the FFQs in different seasons. Table S4. Validity and reliability of the FFQs in different subgroups. (DOCX 39 kb) [file 12937_2019_454_MOESM1_ESM.docx]

| Additional file 1: Table S1 Median and 25–75th percentile(Q1-Q3) of energy and nutrient intake of FFQs among seasons in Shanghai Diet and Health Study | | | | | | | | |
| --- | --- | --- | --- | --- | --- | --- | --- | --- |
|  | Spring |  | Summer |  | Fall |  | Winter |  |
|  | Median | Q1–Q3 | Median | Q1–Q3 | Median | Q1–Q3 | Median | Q1–Q3 |
| Energy(kcal) | 1617.6 | 1383.2-1998.1 | 1566.5 | 1310.1-1869.6 | 1544.6 | 1256.7-1867.2 | 1561.9 | 1280.2-1838.4 |
| Protein(g) | 53.3 | 42.1-66.3 | 54.3 | 42.5-65.8 | 48.4 | 38.5-61.4 | 52.9 | 42.4-64.5 |
| Fat(g) | 57.9 | 43.9-71.7 | 49.8 | 37.2-64.7 | 54.2 | 43.9-67.8 | 47.9 | 34.9-61.9 |
| Carbohydrate (g) | 243.7 | 189.2-289.3 | 227.3 | 180.8-277.9 | 210.0 | 158.2-270 | 228.1 | 182.2-275.2 |
| Protein(% energy) | 12.8 | 11.0-14.5 | 13.7 | 12.0-15.4 | 12.7 | 11.1-14.5 | 13.6 | 11.9-15.4 |
| Fat(% energy) | 31.3 | 25.3-36.4 | 28.6 | 22.7-34.6 | 32.6 | 26.5-38.8 | 27.6 | 21.3-33.6 |
| Carbohydrate (% energy) | 56.7 | 51.1-63.1 | 58.5 | 52.6-64.4 | 55.2 | 48.5-62.1 | 59.5 | 53.2-65.9 |
| Vitamin A(μg) | 301.0 | 205.4-418.1 | 300.3 | 214.9-407.8 | 303.6 | 206.5-408.5 | 301.3 | 213.8-400.2 |
| Carotene(μg) | 866.7 | 548.7-1321.9 | 936.0 | 640.6-1359.2 | 894.0 | 589.1-1301.4 | 951.0 | 634.2-1285 |
| Retinol(μg) | 140.6 | 84.4-204.1 | 133.9 | 83.4-191.2 | 135.7 | 81.0-201.8 | 129.1 | 78.3-189.6 |
| Vitamin D(IU) | 6.5 | 2.2-15.4 | 12.0 | 4.2-23.8 | 7.6 | 2.8-18.2 | 6.0 | 2.2-13.6 |
| Vitamin E(mg) | 23.2 | 17.4-30.2 | 19.7 | 13.1-26.4 | 20.7 | 16.3-27.2 | 17.8 | 10.2-24.9 |
| Vitamin K(μg) | 10.7 | 5.4-21.5 | 8.1 | 4.3-13.7 | 10.7 | 5.4-17.2 | 12.5 | 6.4-21.5 |
| Thiamine(mg) | 0.6 | 0.5-0.8 | 0.6 | 0.5-0.8 | 0.5 | 0.4-0.7 | 0.6 | 0.5-0.8 |
| Riboflavin(mg) | 0.7 | 0.5-1 | 0.7 | 0.5-1 | 0.6 | 0.5-0.9 | 0.7 | 0.5-0.9 |
| Niacin(mg) | 10.9 | 8.5-13.5 | 10.5 | 8.3-13.1 | 9.8 | 7.7-12.5 | 10.8 | 8.7-13.3 |
| Folate (μg) | 11.0 | 6.2-18.2 | 9.9 | 6.1-14.9 | 10.1 | 6.2-15.6 | 10.7 | 7.2-16.8 |
| Biotin(μg) | 0.9 | 0.4-1.6 | 1.1 | 0.5-1.8 | 0.7 | 0.3-1.4 | 0.8 | 0.4-1.4 |
| Choline(mg) | 3.9 | 0.7-12.5 | 6.6 | 2.5-14.6 | 4.2 | 0.3-11.5 | 3.2 | 0.5-10 |
| Vitamin C (mg) | 45.0 | 28.1-65.3 | 46.9 | 32.1-63.7 | 47.3 | 30.6-66 | 47.4 | 32.2-63.8 |
| Calcium (mg) | 357.0 | 232.4-561.9 | 352.6 | 230.6-530 | 337.9 | 216.7-519.4 | 341.3 | 232.9-506.8 |
| Phosphorus (mg) | 777.4 | 605.6-982.5 | 786.8 | 602.5-966.4 | 696.0 | 540.9-887.8 | 761.4 | 602.6-949.7 |
| Potassium (mg) | 1350.4 | 1018.6-1754.8 | 1386.3 | 1029.9-1781.1 | 1240.4 | 924.1-1588.9 | 1313.3 | 1001.3-1660.3 |
| Sodium (mg) | 4034.9 | 2833.1-5214.7 | 3622.8 | 1001.9-5115.5 | 3936.4 | 2688.0-5006.8 | 2950.9 | 638.0-4721.8 |
| Magnesium (mg) | 216.1 | 173.0-269.1 | 212.4 | 166.7-262.7 | 189.9 | 149.0-235.3 | 208.4 | 169.2-255.9 |
| Iron (mg) | 15.6 | 12.3-19.5 | 14.5 | 11.7-18.1 | 13.1 | 10.4-16.3 | 14.1 | 11.6-17.5 |
| Zinc (mg) | 8.8 | 7.1-10.7 | 8.5 | 6.9-10.2 | 8.0 | 6.4-9.9 | 8.5 | 7.0-10.3 |
| Selenium(μg) | 35.4 | 26.2-47.1 | 39.3 | 28.8-49.9 | 36.7 | 26.7-49.5 | 37.5 | 28.8-49.8 |

| Additional file 1: Table S2 Median and 25–75th percentile(Q1-Q3) of energy and nutrient intake of 24-HDR among seasons in Shanghai Diet and Health Study | | | | | | | | |
| --- | --- | --- | --- | --- | --- | --- | --- | --- |
|  | Spring |  | Summer |  | Fall |  | Winter |  |
|  | Median | Q1–Q3 | Median | Q1–Q3 | Median | Q1–Q3 | Median | Q1–Q3 |
| Energy(kcal) | 1656.0 | 1334.3-2000.1 | 1515.2 | 1222.2-1819.7 | 1511.6 | 1251.8-1907.5 | 1562.7 | 1291.9-1883.3 |
| Protein(g) | 60.2 | 45.9-76 | 58.7 | 45.9-74.2 | 54.0 | 42.5-69.7 | 58.9 | 46.1-72.9 |
| Fat(g) | 69.2 | 50.6-89.4 | 60.9 | 46.6-78.8 | 66.5 | 49.7-86.1 | 68.0 | 51.5-87.9 |
| Carbohydrate (g) | 189.1 | 146.7-243 | 173.3 | 134.5-220.3 | 171.5 | 137.8-216.5 | 170.0 | 135.2-213.1 |
| Protein(% energy) | 14.7 | 12.7-17.2 | 15.6 | 13.3-18.1 | 14.2 | 12.2-16.7 | 15.1 | 13.1-17.4 |
| Fat(% energy) | 37.7 | 31.3-45.8 | 37.3 | 30.2-45.4 | 39.5 | 32.6-46.7 | 40.1 | 32.8-47.2 |
| Carbohydrate (% energy) | 47.5 | 40.2-53.9 | 46.8 | 40.1-53.9 | 46.3 | 39.0-52.9 | 44.8 | 38.3-51.3 |
| Vitamin A(μg) | 319.8 | 202.8-467.2 | 361.6 | 242.1-508 | 352.2 | 232.9-533 | 366.4 | 250.5-546.1 |
| Carotene(μg) | 1013.6 | 533.5-1718.3 | 1244.0 | 704.3-2016.8 | 1297.2 | 724.9-2113 | 1316.7 | 780.0-2238 |
| Retinol(μg) | 124.6 | 73.9-185.2 | 131.1 | 84.3-189 | 119.2 | 68.6-181.6 | 116.8 | 69.5-178.1 |
| Vitamin D(IU) | 0.0 | 0.00 | 0.0 | 0.00 | 0.0 | 0.00 | 0.0 | 0.00 |
| Vitamin E(mg) | 27.5 | 18.3-39.5 | 22.2 | 15.6-31.6 | 23.2 | 15.4-34.9 | 22.6 | 15.6-33.3 |
| Vitamin K(μg) | 0.0 | 0.0-52.1 | 0.0 | 0.00 | 0.0 | 0.0-104.2 | 0.0 | 0.00 |
| Thiamine(mg) | 0.7 | 0.5-0.9 | 0.7 | 0.5-0.9 | 0.6 | 0.5-0.8 | 0.7 | 0.5-0.9 |
| Riboflavin(mg) | 0.8 | 0.6-1 | 0.8 | 0.6-1 | 0.7 | 0.5-0.9 | 0.8 | 0.6-1 |
| Niacin(mg) | 13.2 | 10.0-16.9 | 13.1 | 10.1-16.8 | 12.6 | 9.7-16.5 | 14.2 | 11.1-17.5 |
| Folate (μg) | 3.5 | 0.0-42.6 | 2.8 | 0.1-16.2 | 7.7 | 0.1-55.3 | 2.2 | 0.0-7.2 |
| Biotin(μg) | 0.3 | 0.0-1.3 | 0.3 | 0.0-0.9 | 0.4 | 0.0-1.4 | 0.1 | 0.0-0.6 |
| Choline(mg) | 0.0 | 0.0-6.8 | 0.0 | 0.0-1.9 | 0.0 | 0.0-10.2 | 0.0 | 0.0-5.9 |
| Vitamin C (mg) | 49.1 | 29.8-75 | 58.0 | 37.8-83.2 | 61.0 | 38.7-88.1 | 69.1 | 48.4-103.2 |
| Calcium (mg) | 367.9 | 251.7-536.2 | 376.3 | 259.9-548.2 | 383.0 | 260.9-537.3 | 427.0 | 302.6-578.6 |
| Phosphorus (mg) | 843.5 | 662.3-1056.5 | 818.8 | 650.7-994.3 | 774.7 | 616.8-986.9 | 816.0 | 652.8-991.9 |
| Potassium (mg) | 1544.2 | 1164.8-2075.4 | 1495.2 | 1129.0-1924.2 | 1368.4 | 1062.9-1787.3 | 1439.3 | 1107.6-1833.6 |
| Sodium (mg) | 3756.8 | 2669.8-5439.3 | 3914.8 | 2809.7-5504.2 | 3770.0 | 2685.2-5448.2 | 4030.2 | 2872.9-5292.9 |
| Magnesium (mg) | 236.0 | 183.2-300.9 | 221.6 | 173.3-280.1 | 217.5 | 172.9-278.3 | 217.0 | 172.5-270 |
| Iron (mg) | 16.3 | 13.2-20.9 | 16.2 | 13.1-20.9 | 16.0 | 12.7-20.6 | 16.6 | 13.3-21.4 |
| Zinc (mg) | 9.4 | 7.3-12.1 | 8.5 | 6.7-10.4 | 8.4 | 6.8-10.7 | 8.7 | 7.1-10.7 |
| Selenium(μg) | 39.4 | 28.6-53.6 | 43.3 | 30.1-56.8 | 40.2 | 28.7-55.9 | 42.5 | 30.3-56.3 |

| Additional file 1: Table S3 Validity and reliability of the FFQs in different seasons. | | | | | | | | | | |  | |  | | | |  | |
| --- | --- | --- | --- | --- | --- | --- | --- | --- | --- | --- | --- | --- | --- | --- | --- | --- | --- | --- |
|  | | | Validity | | | | | | | | | | reliability | | | | | |
|  | | | Spring | | | Summer | | | Fall | | Winter | | Summer vs Winter | | | | Spring vs Fall | |
| Energy(kcal) | | | 0.64 | | | 0.77 | | | 0.72 | | 0.77 | | 0.71 | | | | 0.59 | |
| Protein(g) | | | 0.36 | | | 0.45 | | | 0.39 | | 0.3 | | 0.56 | | | | 0.72 | |
| Fat(g) | | | 0.42 | | | 0.61 | | | 0.51 | | 0.6 | | 0.69 | | | | 0.62 | |
| Carbohydrate (g) | | | 0.38 | | | 0.39 | | | 0.39 | | 0.36 | | 0.62 | | | | 0.75 | |
| Protein(% energy) | | | 0.64 | | | 0.76 | | | 0.74 | | 0.8 | | 0.72 | | | | 0.59 | |
| Fat(% energy) | | | 0.64 | | | 0.76 | | | 0.73 | | 0.79 | | 0.71 | | | | 0.59 | |
| Carbohydrate (% energy) | | | 0.65 | | | 0.77 | | | 0.74 | | 0.78 | | 0.71 | | | | 0.62 | |
| Vitamin A(μg) | | | 0.55 | | | 0.66 | | | 0.6 | | 0.63 | | 0.56 | | | | 0.55 | |
| Carotene(μg) | | | 0.58 | | | 0.67 | | | 0.62 | | 0.67 | | 0.58 | | | | 0.55 | |
| Retinol(μg) | | | 0.58 | | | 0.7 | | | 0.67 | | 0.71 | | 0.64 | | | | 0.54 | |
| Vitamin D(IU) | | | 0.62 | | | 0.76 | | | 0.72 | | 0.74 | | 0.69 | | | | 0.59 | |
| Vitamin E(mg) | | | 0.59 | | | 0.74 | | | 0.69 | | 0.82 | | 0.79 | | | | 0.64 | |
| Vitamin K(μg) | | | 0.62 | | | 0.73 | | | 0.71 | | 0.76 | | 0.66 | | | | 0.59 | |
| Thiamine(mg) | | | 0.31 | | | 0.35 | | | 0.24 | | 0.21 | | 0.57 | | | | 0.55 | |
| Riboflavin(mg) | | | 0.55 | | | 0.63 | | | 0.58 | | 0.52 | | 0.59 | | | | 0.65 | |
| Niacin(mg) | | | 0.22 | | | 0.4 | | | 0.24 | | 0.25 | | 0.46 | | | | 0.56 | |
| Folate (μg) | | | 0.58 | | | 0.7 | | | 0.68 | | 0.73 | | 0.63 | | | | 0.57 | |
| Biotin(μg) | | | 0.65 | | | 0.75 | | | 0.71 | | 0.71 | | 0.68 | | | | 0.60 | |
| Choline(mg) | | | 0.62 | | | 0.77 | | | 0.72 | | 0.7 | | 0.69 | | | | 0.63 | |
| Vitamin C (mg) | | | 0.56 | | | 0.67 | | | 0.59 | | 0.64 | | 0.56 | | | | 0.53 | |
| Calcium (mg) | | | 0.6 | | | 0.68 | | | 0.64 | | 0.63 | | 0.62 | | | | 0.62 | |
| Phosphorus (mg) | | | 0.45 | | | 0.51 | | | 0.47 | | 0.41 | | 0.61 | | | | 0.78 | |
| Potassium (mg) | | | 0.49 | | | 0.62 | | | 0.47 | | 0.45 | | 0.55 | | | | 0.68 | |
| Sodium (mg) | | | 0.64 | | | 0.76 | | | 0.72 | | 0.81 | | 0.74 | | | | 0.58 | |
| Magnesium (mg) | | | 0.44 | | | 0.48 | | | 0.36 | | 0.37 | | 0.53 | | | | 0.69 | |
| Iron (mg) | | | 0.39 | | | 0.42 | | | 0.37 | | 0.23 | | 0.49 | | | | 0.54 | |
| Zinc (mg) | | | 0.18 | | | 0.33 | | | 0.25 | | 0.16 | | 0.49 | | | | 0.65 | |
| Selenium(μg) | | | 0.48 | | | 0.57 | | | 0.57 | | 0.5 | | 0.60 | | | | 0.58 | |
| Additional file 1: Table S4 Validity and reliability of the FFQs in different subgroups. | | | | | | | | | | | | | | | | | | |
|  | All | | | Men | | | Women | | | 18-44 years | | | | 45-59 years | | >=60 years | | |
|  | FFQ1 versus FFQ2 | 24-HDR  versus FFQ2 | | FFQ1 versus FFQ2 | 24-HDR  versus FFQ2 | | FFQ1 versus FFQ2 | 24-HDR  versus FFQ2 | | FFQ1 versus FFQ2 | | 24-HDR  versus FFQ2 | | FFQ1 versus FFQ2 | 24-HDR  versus FFQ2 | FFQ1  versus  FFQ2 | | 24-HDR  versus FFQ2 |
| Energy(kcal) | 0.71 | 0.76 | | 0.77 | 0.80 | | 0.64 | 0.65 | | 0.78 | | 0.67 | | 0.74 | 0.75 | 0.63 | | 0.81 |
| Protein(g) | 0.55 | 0.44 | | 0.56 | 0.56 | | 0.54 | 0.29 | | 0.67 | | 0.37 | | 0.47 | 0.49 | 0.56 | | 0.47 |
| Fat(g) | 0.70 | 0.61 | | 0.65 | 0.53 | | 0.72 | 0.64 | | 0.71 | | 0.52 | | 0.71 | 0.67 | 0.67 | | 0.62 |
| Carbohydrate (g) | 0.59 | 0.38 | | 0.63 | 0.47 | | 0.55 | 0.30 | | 0.74 | | 0.10 | | 0.65 | 0.46 | 0.50 | | 0.48 |
| Protein(% energy) | 0.73 | 0.76 | | 0.79 | 0.80 | | 0.65 | 0.64 | | 0.79 | | 0.66 | | 0.76 | 0.74 | 0.65 | | 0.81 |
| Fat(% energy) | 0.71 | 0.76 | | 0.77 | 0.79 | | 0.65 | 0.65 | | 0.78 | | 0.67 | | 0.76 | 0.75 | 0.64 | | 0.81 |
| Carbohydrate (% energy) | 0.71 | 0.77 | | 0.76 | 0.80 | | 0.64 | 0.66 | | 0.78 | | 0.68 | | 0.75 | 0.75 | 0.64 | | 0.82 |
| Vitamin A(μg) | 0.60 | 0.68 | | 0.62 | 0.73 | | 0.54 | 0.51 | | 0.58 | | 0.62 | | 0.55 | 0.64 | 0.58 | | 0.68 |
| Carotene(μg) | 0.60 | 0.68 | | 0.61 | 0.72 | | 0.58 | 0.55 | | 0.60 | | 0.63 | | 0.57 | 0.68 | 0.58 | | 0.66 |
| Retinol(μg) | 0.65 | 0.70 | | 0.68 | 0.73 | | 0.59 | 0.56 | | 0.68 | | 0.63 | | 0.66 | 0.66 | 0.61 | | 0.75 |
| Vitamin D(IU) | 0.77 | 0.79 | | 0.60 | 0.74 | | 0.84 | 0.75 | | 0.75 | | 0.67 | | 0.73 | 0.73 | 0.64 | | 0.82 |
| Vitamin E(mg) | 0.76 | 0.73 | | 0.76 | 0.73 | | 0.73 | 0.68 | | 0.85 | | 0.69 | | 0.81 | 0.76 | 0.73 | | 0.72 |
| Vitamin K(μg) | 0.67 | 0.73 | | 0.72 | 0.75 | | 0.62 | 0.62 | | 0.70 | | 0.67 | | 0.68 | 0.70 | 0.65 | | 0.77 |
| Thiamine(mg) | 0.55 | 0.36 | | 0.53 | 0.40 | | 0.57 | 0.30 | | 0.63 | | 0.38 | | 0.54 | 0.39 | 0.58 | | 0.31 |
| Riboflavin(mg) | 0.57 | 0.62 | | 0.59 | 0.69 | | 0.48 | 0.46 | | 0.64 | | 0.60 | | 0.55 | 0.63 | 0.62 | | 0.64 |
| Niacin(mg) | 0.46 | 0.39 | | 0.42 | 0.43 | | 0.51 | 0.37 | | 0.45 | | 0.31 | | 0.45 | 0.46 | 0.50 | | 0.40 |
| Folate (μg) | 0.66 | 0.72 | | 0.71 | 0.74 | | 0.61 | 0.61 | | 0.65 | | 0.64 | | 0.64 | 0.70 | 0.60 | | 0.73 |
| Biotin(μg) | 0.68 | 0.73 | | 0.72 | 0.76 | | 0.62 | 0.61 | | 0.76 | | 0.67 | | 0.69 | 0.73 | 0.64 | | 0.79 |
| Choline(mg) | 0.73 | 0.74 | | 0.87 | 0.76 | | 0.56 | 0.62 | | 0.77 | | 0.68 | | 0.71 | 0.75 | 0.64 | | 0.80 |
| Vitamin C (mg) | 0.55 | 0.65 | | 0.55 | 0.68 | | 0.52 | 0.50 | | 0.56 | | 0.69 | | 0.56 | 0.64 | 0.58 | | 0.68 |
| Calcium (mg) | 0.60 | 0.68 | | 0.65 | 0.74 | | 0.49 | 0.50 | | 0.68 | | 0.63 | | 0.60 | 0.68 | 0.63 | | 0.69 |
| Phosphorus (mg) | 0.59 | 0.51 | | 0.63 | 0.60 | | 0.49 | 0.36 | | 0.72 | | 0.52 | | 0.53 | 0.52 | 0.64 | | 0.51 |
| Potassium (mg) | 0.55 | 0.62 | | 0.52 | 0.62 | | 0.50 | 0.49 | | 0.64 | | 0.61 | | 0.48 | 0.60 | 0.58 | | 0.63 |
| Sodium (mg) | 0.74 | 0.74 | | 0.75 | 0.75 | | 0.70 | 0.66 | | 0.85 | | 0.65 | | 0.80 | 0.72 | 0.60 | | 0.83 |
| Magnesium (mg) | 0.52 | 0.49 | | 0.49 | 0.54 | | 0.52 | 0.35 | | 0.68 | | 0.49 | | 0.49 | 0.48 | 0.49 | | 0.47 |
| Iron (mg) | 0.45 | 0.50 | | 0.45 | 0.54 | | 0.44 | 0.37 | | 0.62 | | 0.38 | | 0.45 | 0.39 | 0.44 | | 0.45 |
| Zinc (mg) | 0.45 | 0.37 | | 0.47 | 0.48 | | 0.44 | 0.24 | | 0.59 | | 0.28 | | 0.46 | 0.36 | 0.42 | | 0.37 |
| Selenium(μg) | 0.61 | 0.57 | | 0.62 | 0.69 | | 0.59 | 0.38 | | 0.67 | | 0.56 | | 0.56 | 0.53 | 0.63 | | 0.61 |
